# Morphological, Molecular and Metabolic Characterization of the Pigmented Fungus *Subramaniula asteroids*

**DOI:** 10.3390/jof8111149

**Published:** 2022-10-29

**Authors:** Heba El-Sayed, Mohamed E. Osman, Asmaa Abdelsalam, Arezue Boroujerdi, Hana Sonbol, Yasmin M. Elsaba

**Affiliations:** 1 Botany and Microbiology Department, Faculty of Science, Helwan University, Cairo 11421, Egypt; 2 Chemistry Department, Claflin University, Orangeburg, SC 29115, USA; 3 Department of Biology, College of Science, Princess Nourah Bint Abdulrahman University, P.O. Box 84428, Riyadh 11671, Saudi Arabia

**Keywords:** *Subramaniula asteroids*, chaetomiaceae, keratitis, metabolic profile, NMR analysis, GC/MS

## Abstract

Chaetomiaceae fungi are ascosporulating fungi whose importance as human pathogens has been frequently ignored. In the current study, a new isolate of the genus *Subramaniula* was described. The fungus was isolated from the soil of Wadi Om Nefa’a, Hurghada in the Red Sea Governorate, Egypt. Previously, *Subramaniula* were misidentified as *Papulaspora* spp. According to molecular analysis, the fungus was identified as *Subramaniula asteroids* OP484336. Remarkably, this species has been found among other fungi responsible for keratitis in humans and has been recorded for the first time in Egypt. Analysing the *Subramaniula asteroids’* metabolic profile was one of the objectives of the current study because little is known about this family’s metabolome. The fungal extract’s untargeted metabolic profiling was carried out by gas chromatography-mass spectroscopy (GC/MS), ^1^H and ^1^H-HSQC nuclear magnetic resonance (NMR) data, and their corresponding databases. In total, fifty-nine metabolites have been reported in the polar and non-polar extracts. The majority of polar metabolites are amino acids and carbohydrates. The non-polar extract’s main components were 1-dodecanamine, N,N-dimethyl-, 1-tetradecanamine, N,N-dimethyl-, and 9-octadecenoic acid ethyl ester. The current study is the first to provide a metabolic profile of *Subramaniula asteroids*, which can be used in chemotaxonomical classification, antifungal drug development, and biological activity investigation of the studied species.

## 1. Introduction

Melanized fungi are significant human pathogens. Human infections have been related to approximately 70 genera of this fungal group [1]. Recently, the importance of Sordariales has been emphasized, especially Chaetomiaceae, whose distribution has been neglected due to identification issues [1]. A significant number of Chaetomium-like fungi fail to form traditional diagnostic structures in culture, making accurate morphological descriptions difficult. In the clinical laboratory, fungi that did not develop conidia were recently labelled as unidentifiable ‘mycelia sterilia’ [2]. *Papulaspora* was used to describe isolates that formed clumps or bulbils, whereas *Madurella* was used to describe filamentous subcutaneous infection strains [1]. Such isolates were observed to be diverse, relating to several genera and families of ascomycetes using molecular phylogenetic DNA sequencing studies. Furthermore, species were discovered that contained numerous other related sub-species that had been previously thought to belong to the same taxon [3]. Despite recent advances in the taxonomy of sterile fungi, *Papulaspora*’s phylogenetic position remains unknown. The occurrence of clumps or papulospores is non-specific and polyphyletic, as a broad diversity of fungi from different genera and orders can produce similar compact masses of cells. Recently, there has been a rise in interest in nonsporulating chaetomium-like isolates that are an infection source in humans similar to keratitis or subcutaneous infection after trauma [4]. Despite using recent molecular techniques, researchers were unable to categorize these isolates in the genus *Chaetomium* at the species level due to the genus’ current state of morphological confusion and significant phylogenetic variation. Over 300 *Chaetomium* species have been described, and although most lack current descriptions, only a few have been defined with DNA data. For the taxonomic analysis of fungal species, the chemotaxonomic method using metabolic profiling is a high-throughput technology that supplements current molecular methods [5]. NMR structure analysis is a useful tool for studying metabolic profiling of living organisms. Because of its distinctive characteristics, including universal, repeatable, and non-destructive measurements, and the quick development of NMR software and hardware, metabolomic researchers are now able to use this tool for the immediate detection and quantification of compounds present inside any living system [6]. NMR analysis is increasingly being used in pathogenic fungus metabolic profiling. It provides accurate information on metabolites found inside the tissue as well as those produced externally, which can help differentiate between fungi of the same genus [7]. GC/MS is ideal for the investigation of the non-polar metabolites. Because of its high level of sensitivity, GC-MS may be used to analyze compounds that are present in minute concentrations [8].

The primary goal of the current study is to describe a novel fungal strain isolated from Hurghada, Wadi Om Nefa’a, Red Sea Governorate, Egypt, using molecular characterization, which was previously erroneously morphologically identified as *Papulaspora* species [8]. The metabolic profile of the new isolate was investigated using NMR spectroscopy and GC/MS.

## 2. Materials and Methods

### 2.1. Collection of the Studied Soil Samples

**Figure 1:**
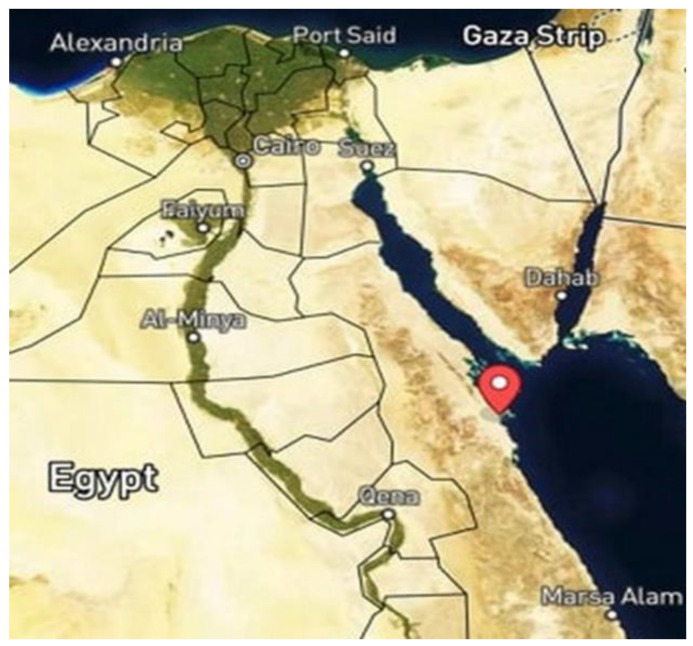
Map showing the location of sample collection.

The enriched soil around cultivated plants such as *Zilla spinosa, Artemisia Judaica, Cleome droserifolia*, *Lotus* sp., and *Acacia raddiana* was collected from Wadi Om Nefa’a, Hurghada, Red Sea Governorate (27°4′13.07″ N, 33°24′29.30″ E), Egypt (Figure 1), in the summer of 2018. The soil was placed in the Mycology lab, Faculty of Science, Helwan University, Egypt.

### 2.2. Fungal Isolation and Morphological Characterization

The studied strain was isolated using the serial dilution method [9]. Serial dilutions were obtained by mixing a 1 g soil sample with sterilized distilled water (9 mL) in a sterilized test tube, shaking well, and then transferring 1 mL of the soil suspension to achieve a serial dilution of 10^−3^. Then, each dilution was transferred to a Petri dish with potato dextrose agar medium (PDA), then rotated to mix the soil dilution with the media before solidification of the media. The cultured Petri dishes were then stored at 25 °C for 7 days. The isolated fungal species was purified and morphologically identified as *Papulaspora irregularis* at Mycological Center, Assiut-University, Egypt (AUMC).

### 2.3. Submerged Cultivation of the Fungal Isolate

A 50 mL potato dextrose yeast extract (PDY) broth medium containing 1 L potato infusion, 20 g dextrose, and 2 g yeast extract [10] was inoculated with two fungal plugs (5 mm) of one-week-old PDA fungal culture. The fungal cultivation was performed using static incubation conditions for 7 days at 25 °C. The fungal biomass and culture filtrate were separated by filtration using Whatman filter paper. The mycelia were thoroughly washed with distilled water before being lyophilized and stored for further analysis.

### 2.4. Molecular Identification

#### 2.4.1. Extraction of Fungal DNA and Polymerase Chain Reaction (PCR) Amplification

The total genomic DNA was extracted from the fungal mycelium of one-week-old PDA culture using Quick-DNA™ Fungal/Bacterial Miniprep Kit (ZYMO RESEARCH). *COSMO* PCR RED Master Mix (W1020300X) (Birmingham, England) was used for the PCR reactions according to manufacture instructions. All reactions were conducted in a reaction volume of 50 µL, with the internal transcribed spacer 1 (ITS1) and ITS4 primers used to amplify ribosomal internal transcribed spacer. The purified product using Zymo-Spin™ Technology was sequenced with Sanger technology using an ABI 3730xl DNA sequencer and ITS4 primer from Eurofins Genomics (formerly GATC Biotech; Ebersberg, Germany).

#### 2.4.2. Sequencing and Analysis

The blast was carried out for the achieved nucleotide sequence using Geneious pro program 11.1.5. Comparatively, the sequence obtained from the National Center for Biotechnology Information (NCBI) (https://blast.ncbi.nlm.nih.gov/Blast.cgi, accessed on 20 January 2019), with high similarity, was aligned using MUSCLE tools. The phylogenetic tree was constructed using maximum likelihood methods [11].

#### 2.4.3. Polar and Non-Polar Fungal Metabolites Extraction

Liquid nitrogen was used to ensure a uniform consistency of the dry biomass. Metabolites were extracted using a 2:2:1.8 ratio of methanol, chloroform, and water based on the dry weight of the sample [12]. The amount of solvent that should be applied to each sample was calculated using the dry mass and the percentage of water loss [13,14]. Cold methanol and water were added to each 20 mg sample, and the mixture was vortexed before being added to cold chloroform and water in glass tubes. The solution was placed in an ice bath for 10 min before being centrifuged at 4 °C for 10 min at 2000× *g*. The top layer (polar) was separated and dried in a Centrivap vacuum concentrator (Labconco, Kansas City, MO, USA) for approximately 48 h until the polar solvents were entirely evaporated, while the non-polar layer was transferred to fresh glass vials and dried using nitrogen gas.

#### 2.4.4. NMR Data Collection and Metabolite Identification

To acquire NMR data and process the mycelium polar extract, the following procedures were followed. Each sample was reconstituted in 620 μL NMR buffer composed of 100 mM sodium phosphate buffer (pH 7.3), 1 mM TMSP (internal standard,3-(trimethylsilyl) propionic-2,2,3,3-d4 acid), and 0.1% sodium azide, in D_2_O). The 1D and 2D data were collected using a 700 MHz Bruker Avance^TM^ III spectrometer. The data were gathered in 2.9 s using 64K points and a spectral width of 16.0 ppm. Overall, 120 scans, 4 dummy scans, 3 s of relaxation delay, and on-resonance pre-saturation at the residual water frequency were utilized to get the first increment of the presat-noesy spectra for solvent suppression. Topspin 2.1.1′s automatic pulse calculation experiment (pulsecal) was used to calculate the pulse widths at 90 °C for each sample (BrukerBioSpin, Billerica, MA, USA). The Bruker hsqcedetgpsisp 2.2-pulse sequence was used to collect two-dimensional HSQC data. The ^1^H was observed in the F2 channel with a spectrum width of 11 ppm, whereas the ^13^C was detected in the F1 channel with a spectral width of 180 ppm.

The fungal metabolites of the polar extract were identified by comparing our ^1^H data to the Chenomx NMR Suite’s chemical library (Chenomx Inc., Edmonton, Alberta, Canada). To confirm the identities of the metabolites obtained by Chenomx, ^1^H^−13^C HSQC results were compared to reference data from the Human Metabolome Database (HMDB) (https://hmdb.ca/, accessed on 23 October 2022) and the scientific literature.

#### 2.4.5. GC/MS Data and Metabolite Identification

The non-polar extract of the fungal mycelium was analyzed with a TRACE GC Ultra Gas Chromatograph instrument (Thermo Fisher Scientific, Waltham, MA, USA) equipped with both a thermal mass spectrometer detector (ISQ Single Quadrupole Mass Spectrometer) and a TR-5 MS column (30 m × 0.32 mm i.d., 0.25 µm film thickness). The experiment was conducted with helium serving as the carrier gas at a flow rate of 1.0 mL/min and a split ratio of 1:10. After setting it at 60 °C for one min, the temperature was ramped at a rate of 4 °C/min to 240 °C and then maintained for one minute. Both the injector and the detector were kept at a temperature of 210 °C. The sample was diluted 1:10 with hexane (*v*/*v* before being injected as 1.0 µL aliquot. The mass spectral range was set to *m*/*z* 40–450; electron ionization (EI) was performed at 70 eV. The identification of the non-polar metabolites was performed using the AMDIS program (www.amdis.net, accessed on 23 October 2022), retention indices (relative to n-alkanes C8-C22), and mass spectrum, which were matched to authentic standards (once it becomes accessible) as well as NIST databases and Wiley spectral library.

## 3. Results

**Figure 2:**
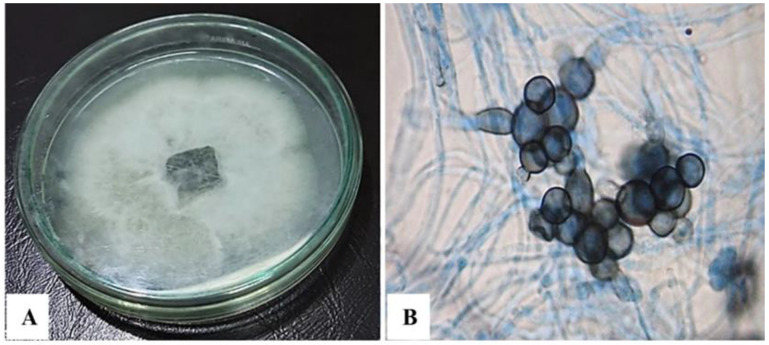
The morphological (**A**) and microscopic (**B**) characterization of *Subramaniula asteroids*.

The fungal morphological examination was performed on potato dextrose agar (PDA). Within three days of incubation, the colonies were white and cottony, then gradually dark pigmentation was observed at the reverse surface (Figure 2A). Microscopic examination showed the cellular clump formation, which is typical of *Papulaspora* species (Figure 2B).

**Figure 3:**
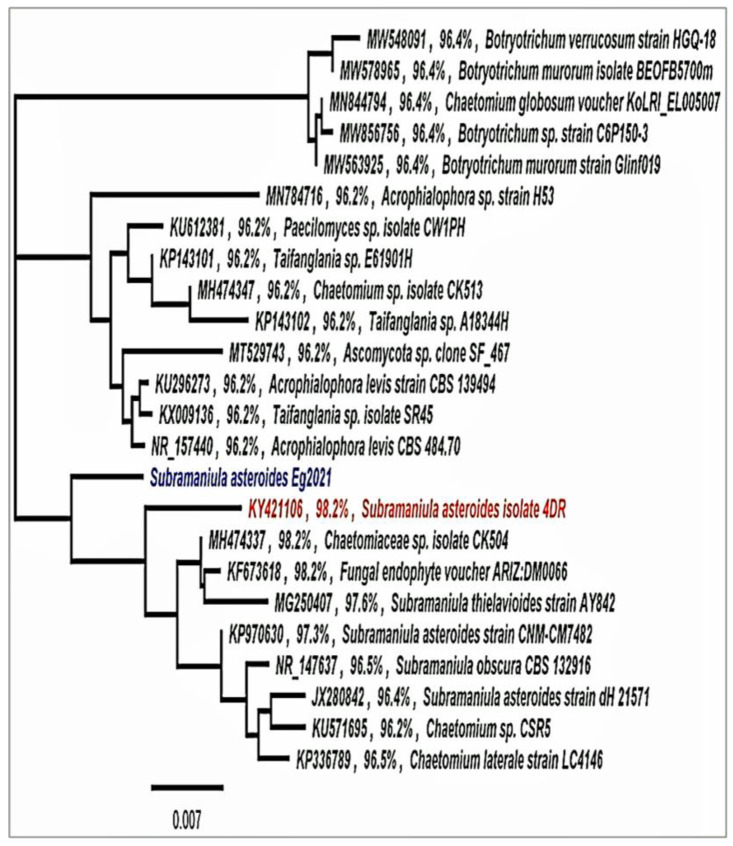
The molecular characterization of *Subramaniula asteroids*. The phylogenetic tree was drawn by Geneious pro program using maximum likelihood methods.

Subsequently, the fungal DNA nucleotide sequence was examined using advanced Basic Local Alignment Search Tool (BLAST) (Megablast) searches from the National Center for Biotechnology Information in the GenBank database (NCBI). The fungal isolate was identified and submitted to the GenBank as *Subramaniula asteroids* Eg2021 with accession number OP484336. In addition, a phylogenetic tree constructed from the partial sequence and related sequences from other fungi in GenBank demonstrated that *Subramaniula asteroids* Eg2021 is 98.2% similar to *Subramaniula asteroids* 4DR (Figure 3).

### NMR Spectroscopy and GC/MS-Based Metabolic Profiling

*Subramaniula asteroids* polar extract was explored in this study by analyzing 1D NMR spectra using the Chenomx database, then com paring 2D NMR data with those found in the HMDB and literature.

**Figure 4:**
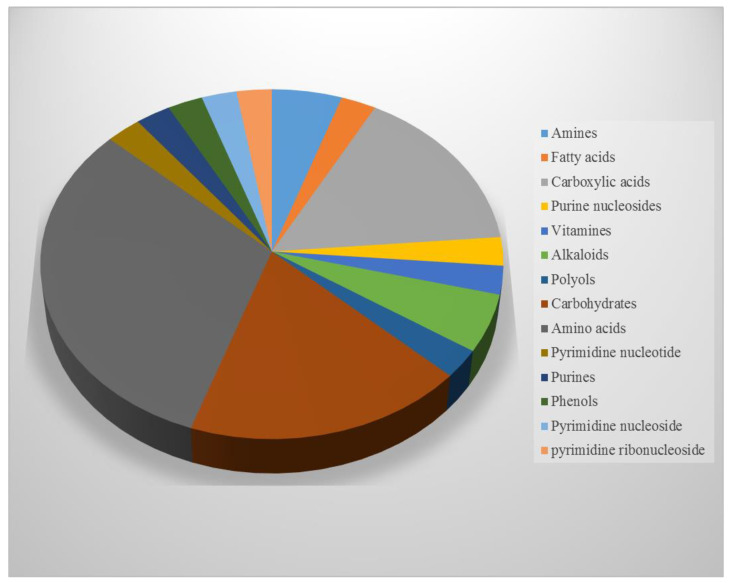
The percentage of chemical classes identified in the polar extract of *Subramaniula asteroids*.

**Figure 5:**
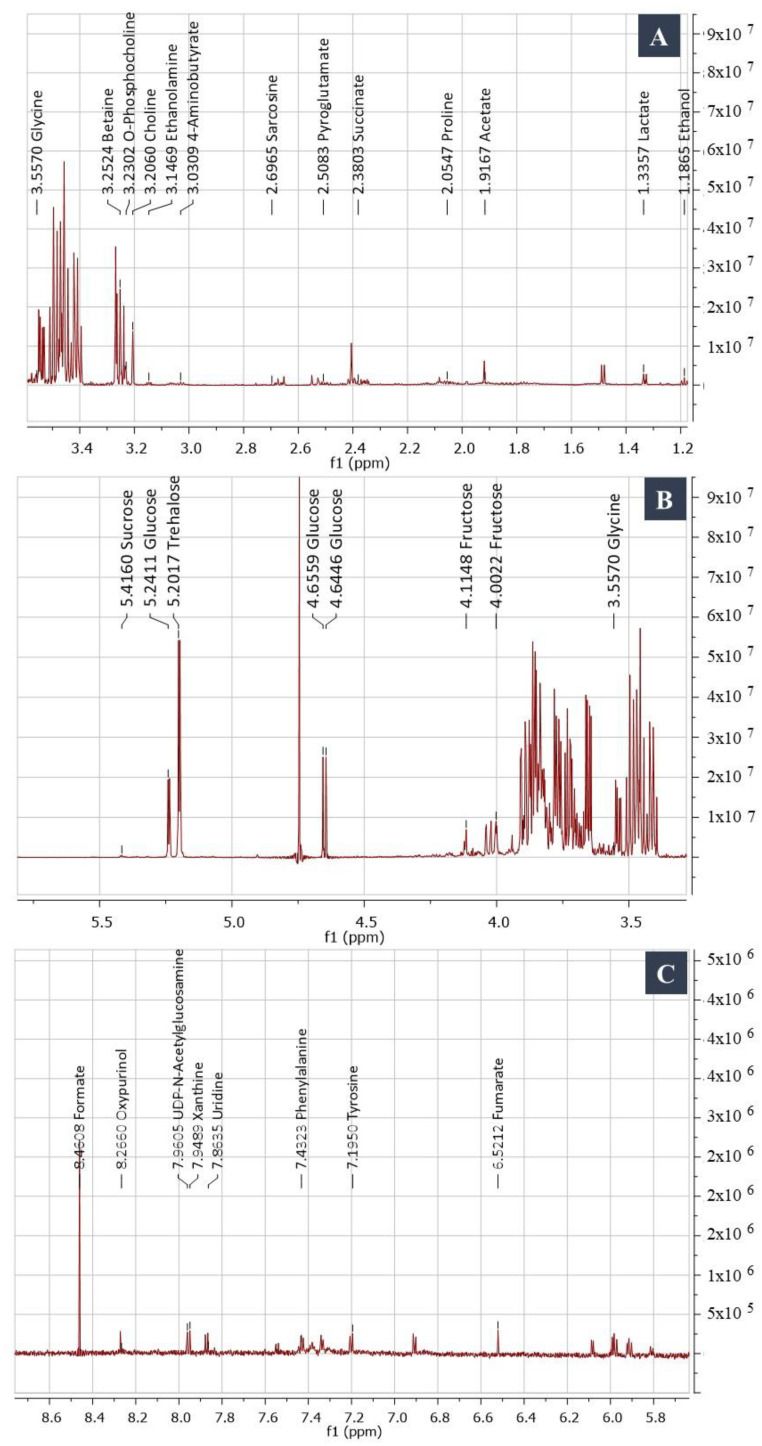
1D ^1^H NMR spectra of *Subramaniula asteroids* polar extract. (**A**) Aliphatic region, (**B**) sugar abundance signals region, and (**C**) aromatic region.

Of the polar compounds, 32% are amino acids, 19% are sugars, 5.7% are alkaloids, 3% are phenols, and 2.7% are vitamins (Figure 4). The cross peaks observed in the HSQC spectrum facilitated the assignment of ^1^H-^13^C correlation. The amino acids alanine and valine were identified by doublet signals at δH 1.48 ppm (3H^β^, J = 7.25 Hz) correlated with δC 18.6 and δH 1.03 (3H^γ^, J = 7.00 Hz) and correlated with δC 19.5, respectively; arginine was identified with signals at δH 1.64 (2H^γ^, m), δH 1.92 (2H^β^, m), and δH 3.75 (1H^α^, t, J = 6.2 Hz) (Figure 5A). Sugar abundance regions were characterized by the presence of glucose, fructose, sucrose, and trehalose signals (Figure 5B). The monosaccharides glucose and fructose showed signals attributed to their anomeric protons. Additionally, the characteristic resonance positions of these protons and their corresponding coupling constants distinguished the two configurations of glucose as α- (δH 5.24, d, J = 3.68 Hz)/δC 98.9 and β- (δH 4.66, d, J = 7.88 Hz)/δC 94.8. Sucrose and trehalose were identified by the proton signal at δH 5.42 (1H, d, J = 3.89 Hz)/δC 95.1; and δH 5.20 (1H, d, J = 3.73 Hz)/δC 95.8, respectively. The 1H NMR spectrum revealed the presence of three signals with multiplet splitting in the aromatic region (Figure 5C) at δH 7.31, 7.36, and 7.41 that were assigned for the aromatic amino acid phenylalanine. Moreover, tyrosine identified by the multiplet split signals resonated at δH 6.88 and 7.18; 3.10 (dd, J = 7.30, 14.30 Hz), respectively. Alkaloids oxypurinol and xanthine have been annotated based on the two singlet signals at δH 8.2 and 7.9, respectively.

As shown in Table 1, the polar metabolites have been described with their chemical formula, molecular weight, coupling constants, and 1D and 2D NMR chemical shifts.

GC/MS was utilized to characterize the chemical profile of the non-polar extract. Twenty-one metabolites were successfully identified: the main constituents were 1-dodecanamine, N,N-dimethyl-, 1-tetradecanamine, N,N-dimethyl-, and 9-octadecenoic acid ethyl ester, with peak areas of 27.96, 10.10, and 9.27%, respectively. Metabolites cyclopropanebutanoic acid, 2-[[2-[[2-[(2-pentylcyclopropyl) methyl] cyclopropyl] methyl] cyclopropyl]; phen-1,4-diol, 2,3-dimethyl-5-trifluoromethyl-methyl], methyl ester and 7,9-di-tert-butyl-1-oxaspiro (4, 5) deca-6,9-diene-2,8-dione were represented in relatively lower concentrations, with peak areas of 0.91, 0.92, and 0.93%, respectively. The non-polar metabolites are recorded in Table 2 along with their chemical formula, molecular weight, retention time, and peak area (%).

## 4. Discussion

In the current investigation, a new fungal strain of *Subramaniula asteroids* was reported. The fungus was isolated from the unique Red Sea soil habitat in Egypt. The fungus was erroneously identified as *Papulaspora* species through morphological examination [1,3]. The fungus was identified as *Subramaniula asteroids* OP484336 using molecular analysis. The fungal isolate, which was known as a member of the family Cheatomiaceae, has been recently reported as one of the new keratitis causative agents [15], and has been recorded for the first time in Egypt. Keratitis is a clinical illness that causes irritation of the eye’s cornea and is one of the primary causes of monocular blindness globally. Ocular surface disease, trauma, immunocompromised state, and contact lens usage are the major individual risk factors for keratitis [16,17]. Various microbes have the ability to cause keratitis, including viruses [18], protozoa [19], bacteria [20], and fungi [21]. Fungi keratitis contributes to 40–50% of all microbial keratitis infections [22]. However, diagnosing fungal keratitis is more difficult because a variety of fungal pathogens can cause the same disease symptoms, including *Candida*, *Fusarium*, and *Aspergillus* sp. [23]. Filamentous fungi and yeasts can both cause fungal keratitis. In tropical regions, filamentous fungal infections are prevalent [15,24]. Otherwise, yeasts are most common in countries with temperate climates [24]. One of the goals of the current study was to investigate the metabolic profile of the *Subramaniula asteroids* family, as little is known about their metabolome.

The metabolic fingerprint of the fungus generated with analytical instrumentation provides a high-precision platform for obtaining accurate information on a wide range of metabolites produced by the organism [15,16,17,18,19,20,21,22,23,24]. It can be used to give perspective into a fungal species’ biological activities, as well as play a role in taxonomic identification [6,25,26,27,28].

In the present study, NMR and GC/MS data and databases were used to determine the metabolic profile of *Subramaniula asteroids* polar and non-polar extracts. The chemical profiling of the fungal mycelium showed the presence of chemical compounds with reported biological activities. Thirty-eight metabolites were detected in the fungus polar extract based on ^1^H and ^1^H-^13^C HSQC NMR data and NMR databases. Using combined ^1^H and ^1^H-^13^C HSQC NMR data to examine the metabolic profile of living organisms is an accurate and efficient method [29]. The identified polar metabolites included twelve amino acids (e.g., arginine, proline, and betaine) and seven carbohydrates (e.g., trehalose, sucrose, and glucose). Amino acids and carbohydrates are important virulence factors in the pathogenicity of human pathogenic fungus [30]. The production of trehalose has been reported in various human pathogenic fungi and its accumulation has been associated with stress conditions, causing metabolism alternation and cell wall instability [31,32]. Trehalose is a critical metabolite during fungi–host infection; for example, by altering the genes responsible for trehalose production, *Candida albicans* infection was reduced [33,34]. In addition, in *Cryptococcus neoformans,* trehalose production must be consistent in order for pathogenicity to occur [35]. The essential role of amino acids in pathogenicity of fungi has been reported [36]. Arginine, proline, and ornithine are essential for yeast-to-hyphal conversions in the human pathogen *Candida albicans* [37].

Herein, two alkaloids (oxypurinol and xanthine) have been identified in the spectral regions δ (7.5–8.5 ppm) and have been reported for further biological activities and medicinal uses. Oxypurinol has antioxidant activity [38] and it has been shown to enhance coronary and peripheral endothelial function in patients suffering from coronary artery disease [39]. Xanthine and its derivatives have been shown to have anticancer, anti-inflammatory, and antibacterial effects [40].

In the present study, twenty metabolites were identified in the non-polar extract using GC/MS. Most of these metabolites possess biological activities that have been reported in the literature. The main component was 1-Dodecanamine, and this metabolite has been reported to possess antioxidant and antimicrobial activities [41]. There were also significant amounts of 9-octadecenoic acid ethyl ester, oleic acid, and n-Hexadecanoic acid in the fungal extract. The antimicrobial activity of 9-octadecenoic acid ethyl ester against *Candida albicans*, *Bacillus subtilis*, *Pseudomonas aeruginosa*, *Escherichia coli*, and *Staphylococcus aureus* has been reported by [42]. n-Hexadecanoic acid has been shown to have anti-inflammatory, anti-rheumatic, and anticancer effects [43,44]. Oleic acid is a type of unsaturated fatty acid that has several health advantages, such as anti-cancer and anti-inflammatory characteristics, as well as effects on the immune system and skin healing [45], and showed antibacterial activity against *K. pneumonia* [5]. The present data reported a small amount of the compound Phen-1,4-diol, 2,3-dimethyl-5-trifluoromethyl: this compound exhibited antioxidant, anti-tuberculosis, and anti-clotting properties [46].

These results provide a morphological, molecular, and chemical characterization of *Subramaniula asteroids*, one of the keratitis-causing microbes. This information will be useful for learning more about the fungus and may play an important role in the fungus management of keratitis patients infected with this fungus.

## 5. Conclusions

A new strain of the keratitis pathogen *Subramaniula asteroids* was isolated from Hurghada, Wadi Om Nefa’a, Red Sea Governorate, Egypt. Morphological and molecular techniques were used to correctly identify the fungus. The current work is the first to provide a metabolic profile of *Subramaniula asteroids*, which can be employed in the species’ chemotaxonomical classification, antifungal drug development, and biological activity assessment.

## Figures and Tables

**Table 1:** The identified metabolites in the polar extract of *Subramaniula asteroids*.

	Compound Name	Chemical Formula	Weight (Da)	Assigned ^13^C Chemical Shift from ^1^H-^13^C HSQC (2D NMR)	Assigned ^1^H Chemical Shift from ^1^H (1D NMR)	Coupling Constant
1	3-Hydroxyisovalerate	C_5_H_10_O_3_	118.13	30.6 (CH_3_) 52.0 (CH_2_)	1.26 (CH_3_, s) 2.35 (CH_2_, s)	- -
2	4-Aminobutyrate	C_4_H_9_NO_2_	103.11	26.4 (CH_2_) 37.0 (CH_2_) 42.0 (CH_2_)	1.90 (CH_2_, m) 2.28 (CH_2_, t) 2.99 (CH_2_, t)	- 7.35 7.40
3	Acetate	C_2_H_4_O_2_	60.05	26.2 (CH_3_)	1.92 (CH_3_, s)	-
4	Alanine	C_3_H_7_NO_2_	89.09	53.5 (C^α^) 18.9 (C^β^)	3.78 (H^α^, q) 1.46 (H^β^, d)	7.2, 7.26
5	Arginine	C_6_H_14_N_4_O_2_	174.20	26.6 (C^γ^) 30.3 (C^β^) 57.1 (C^α^)	1.64 (H^γ^, m) 1.92 (H^β^, m) 3.75 (H^α^, t)	- - 6.2
6	Betaine	C_5_H_11_NO_2_	117.14	69.2 (CH_2_) 56.2 (CH_3_)	3.90 (H^α^, s) 3.26 (H^β^, s)	- -
7	Choline	C_5_H_14_NO	104.17	58.6 (C^α^) 56.8 (C^γ^) 70.4 (C^β^)	4.06 (H^α^_,_ m) 3.21 (H^γ^, s) 3.52 (H^β^, m)	- - -
8	Ethanol	C_2_H_6_O	46.06	119.5 (CH_3_) 60.3 (CH_2_)	1.17 (CH_3_, t) 3.63 (CH_2_, q)	6.7 6.7
9	Ethanolamine	C_2_H_7_NO	61.08	60.5 (CH_2_) -	3.8 (CH_2_, t) 3.13 (CH_2_, t)	5.21
10	Formate	CH_2_O_2_	46.02	-	8.44 (CH, s)	_-_
11	Fructose	C_6_H_12_O_6_	180.15	78.2 (^3^CH) 66.1 (^6^CH_2_) 72.0 (^5^CH) 66.6 (^1^CH)	4.12 (^3^CH, dd) 4.02 (^6^CH_2_, dd) 4.00 (^5^CH, m) 3.56 (^1^CH, m)	12.72, 1.04 7.7, 5.4
12	Fumarate	C_4_H_4_O_4_	116.07	138.0 (CH)	6.49 (CH, s)	-
13	Gallate	C_7_H_6_O_5_	170.11	111.8 (CH)	7.00 (CH, s)	-
14	Glucose	C_6_H_12_O_6_	180.15	98.9 (^1β^CH) 94.8 (^1α^CH) 74.4 (^2α^CH) 72.6 (^4^CH) 63.7 (^6^CH)	4.66 (^1β^CH, d) 5.24 (^1α^CH, d) 3.53(^2α^CH, m) 3.41 (^4^CH, m) 3.74 (^5β^CH, m)	7.88 3.68 - - -
15	Glucose-6-P	C_6_H_13_O_9_P	260.13	94.9 (^1α^CH) 98.9 (^1β^CH) 65.4 (^5β^CH)	5.2 (^1α^CH, d) 4.6 (^1β^CH, d) 4.0 (^5β^CH, m)	3.75 8.0 -
16	Glutamate	C_5_H_9_NO_4_	147.12	29.7 (C^β^) 36.2 (C^γ^) 57.3 (C^α^)	2.04 (H^β^, m) 2.35 (H^γ^, m) 3.74 (H^α^, dd)	- - 7.1, 4.7
17	Glycine	C_2_H_5_NO_2_	75.06	44.3 (C^α^)	3.56 (H^α^, s)	-
18	Isoleucine	C_6_H_13_NO_2_	131.17	26.8 (C^γ^) 17.5 (C^γ^)	1.29 (H^γ^, m) 1.00 (H^γ^, d) 0.91 (H^δ^, t)	- 7.0 7.1
19	Lactate	C_3_H_6_O_3_	90.07	22.1 (CH_3_)	1.33 (CH_3_, d)	6.88
20	Leucine	C_6_H_13_NO_2_	131.17	24.7 (C^δ^)	0.98 (H^δ^, t)	6.1
22	Methylsuccinate	C_5_H_8_O_4_	132.11	19.7 (CH_3_) 44.9 (CH_2_)	1.01 (CH_3_, d) 2.11 (CH_2_, m)	6.7
23	O-Phosphocholine	C_5_H_15_NO_4_P	184.15	56.5 (CH_3_) 86.9 (CH_2_) 60.6 (CH_2_)	3.20 (CH_3_, s) 3.58 (CH_2_, m) 4.15 (CH_2_, m)	- - -
24	Oxypurinol	C_5_H_4_N_4_O_2_	152.11	-	8.20 (CH, s)	-
25	Phenylalanine	C_9_H_11_NO_2_	165.18	39.2 (C^β^) 58.6 (C^α^) 132.1(C^δ^) 130.4(C^ζ^) 131.8(C^ε^)	3.10 (H^β^, dd) 3.39 (H^α^, q) 7.31 (H^δ^, m) 7.36 (H^ζ^, m) 7.41(H^ε^, m)	15.4,6.3
26	Proline	C_5_H_9_NO_2_	115.13	64.2 (C^α^) 49.0 (C^δ^) 31.9 (C^β^) 26.7 (C^γ^)	4.13 (H^α^, m) 3.41 (H^δ^, m) 2.36 (H^β^, m) -	- -
27	Pyroglutamate	C_5_H_7_NO_3_	129.11	27.9 (C^β^) 60.9 (C^α^)	2.40 (H^γ^,m) 4.16 (H^α^, dd)	9.0, 5.9
29	Sarcosine	C_3_H_7_NO_2_	89.09	35.5 (CH_3_) 53.4 (CH_2_)	2.73 (CH_3_, s) 3.61 (CH_2_, s)	- -
31	Succinate	C_4_H_6_O_4_	118.08	37.1 (CH_2_)	2.41 (CH_2_, s)	-
32	Sucrose	C_12_H_22_O_11_	342.29	95.1 (^1^CH) 79.8 (^3′^CH) 76.8 (^4′^CH) 73.9 (^2^CH) 72.1 (^3^CH) 65.3(^6^CH_2_) 64.2 (^1′^CH_2_) 72.1 (^3^CH)	5.42 (^1^CH, d) 4.22 (^3′^CH, d) 4.06 (^4′^CH, t) 3.56 (^2^CH, m) 3.48 (^3^CH, m) 3.83 (^6^CH_2_, m) 3.69 (^1′^CH_2_, s) 3.48 (^3^CH, m)	3.89 8.8 38.6
34	Trehalose	C_12_H_22_O_11_	342.30	95.8 (^1^CH) 73.6 (^2^CH)	5.20 (^1^CH, d) 3.66 (^2^CH, dd) 3.46 (CH, t)	3.73 10, 3.97 9.54
35	Tyrosine	C_9_H_11_NO_3_	181.18	38.2 (C^β^) 118.9(C^ε^) 133.9(C^δ^)	3.10 (H^β^, dd) 6.88 (H^ε^, m) 7.18 (H^δ^, m)	7.30, 14.30
36	UMP	C_9_H_13_N_2_O_9_P	324.18	105.2 (CH) 90.9 (CH)	5.97 (CH, m) 5.98 (CH, m)	- -
37	Uridine	C_9_H_12_N_2_O_6_	244.20	72.0 (CH) 92.0 (CH)	4.21 (CH, dd) 5.90 (CH, d)	9.0, 4.3 9.0
38	Valine	C_5_H_11_NO_2_	117.14	63.0 (C^α^) 20.8 (C^γ^) 19.5 (C^γ^)	3.60 (H^α^, d) 0.98 (H^γ^, d) 1.03 (H^γ^, d)	4.54 7.00 7.00
39	Xanthine	C_5_H_4_N_4_O_2_	152.11	140.3 (CH)	7.90 (CH, s)	-

**Table 2:** The metabolites detected in the non-polar extract of *Subramaniula asteroids*.

	Compound Name	Chemical Formula	Weight (Da)	RT (min)	Area %
1	Tetrahydro-2-(12-pentadecynyloxy)-2H-pyran	C_20_H_36_O_2_	308	7.20	1.56
2	1-Dodecanamine, N,N-dimethyl-	C_14_H_31_N	213	11.95	27.96
3	2,4-Di-tert-butylphenol	C_14_H_22_O	206	12.22	1.30
4	1-Tetradecanamine, N,N-dimethyl-	C_16_H_35_N	241	15.75	10.10
5	2H-Pyran-3-ol, tetrahydro-2,2,6-trimethyl-6-(4-meth yl-3-cyclohexen-1-yl)-, [3S-[3à,6à(R*)]]	C_16_H_26_O_2_	250	16.55	1.30
6	Phen-1,4-diol, 2,3-dimethyl-5-trifluoromethyl-	C_9_H_9_F_3_O_2_	206	18.64	0.92
7	7,9-Di-tert-butyl-1-oxaspiro(4,5)dec a-6,9-diene-2,8-dione	C_17_H_24_O_3_	276	19.51	0.93
8	Cyclopropanebutanoic acid, 2-[[2-[[2-[(2-pentylcyclopropyl)meth yl] cyclopropyl]methyl]cyclopropyl] methyl]-, methyl ester	C_25_H_42_O_2_	374	19.61	0.91
9	n-Hexadecanoic acid (palmitic acid)	C_16_H_32_O_2_	256	20.28	4.13
10	Hexadecanoic acid, ethyl ester	C_18_H_36_O_2_	284	20.72	7.05
11	9-Octadecenoic acid (Z)-, methyl ester	C_19_H_36_O_2_	296	22.37	1.41
12	1-(N-Benzyl-N-methylamino)-4-methoxybutan-2-one	C_13_H_19_NO_2_	221	22.55	4.68
13	Oleic acid	C_18_H_34_O_2_	282	23.03	4.85
14	Ethyl (9E,12E)-octadeca-9,12-dienoate	C_20_H_36_O_2_	308	23.30	8.31
15	Ethyl Oleate	C_20_H_38_O_2_	310	23.39	9.27
16	octadecenoic acid ethyl ester	C_20_H_40_O_2_	312	23.79	1.94
17	(1RS,2RS,1’SR)-2-[(Dimethylamino) phenylmethyl] cycloctanol	C_17_H_27_NO	261	25.60	1.25
18	Squalene	C_30_H_50_	410	32.05	6.42
19	9(11)-Dehydroergosterol tosylate	C_35_H_48_O_3_S	548	33.57	2.09
20	Glycidyl oleate	C_21_H_38_O_3_	338	35.96	1.49

## Data Availability

All data generated or analyzed during this study are included in this article.
